# Drug-associated abdominal aortitis and retroperitoneal fibrosis after treatment with nivolumab

**DOI:** 10.1016/j.jvscit.2025.101824

**Published:** 2025-04-28

**Authors:** Naomi Wedel, Benjamin Wajda, Richard Cormack, Kenton Rommens

**Affiliations:** a Libin Cardiovascular Institute, Calgary, Alberta, Canada; b Division of Vascular Surgery, University of Calgary, Calgary, Alberta, Canada; c Division of Orthopedic Surgery, University of Calgary, Calgary, Alberta, Canada; d Division of Interventional Radiology, University of Calgary, Calgary, Alberta, Canada; e Calgary Aortic Program, Calgary, Alberta, Canada

**Keywords:** Aortitis, Retroperitoneal fibrosis, Immunotherapy, Immune checkpoint inhibitors

## Abstract

A 60-year-old man with metastatic supraglottic laryngeal cancer treated with nivolumab, an immune checkpoint inhibitor (ICI), developed abdominal aortitis and retroperitoneal fibrosis. The nivolumab was discontinued and no steroids were initiated. A literature search found 19 other cases of ICI-associated aortitis. The evidence is limited and conflicting on the optimal management of these rare but serious adverse events. With increasing application in the treatment of a multitude of cancer types, physicians must remain aware of aortitis as an adverse event and consider the potential risks vs benefits before initiating ICI treatment for their patient.

Immune checkpoint inhibitors (ICIs) are immunotherapy drugs for treatment of a variety of cancer types.^1^ These monoclonal antibodies inhibit immune checkpoint molecules to provide antitumor activity.^2^ Despite the clinical benefits, ICIs are associated with immune-related adverse events (irAEs), which are considered off-target tissue damage and involve essentially every organ system.^2^ Vasculitis is a rare toxicity associated with ICIs, with an estimated incidence of 0.26% and affecting vessels of any size.^3^^,^^4^ Aortitis is considered a very rare irAE and carries the risk of aneurysmal degeneration and rupture that can be life threatening.^3^ This case report describes a patient with metastatic supraglottic laryngeal cancer treated with nivolumab, an ICI, who subsequently developed abdominal aortitis and retroperitoneal fibrosis. The patient consented to publication of their case and images.

## Case report

A 60-year-old man was diagnosed with stage IVB squamous cell carcinoma of the supraglottic larynx in June 2020. He had a past medical history of rectal adenocarcinoma treated with neoadjuvant chemoradiation and abdominoperineal resection in 2013, hypothyroidism, and active smoking. The tumour was positive for expression of PD-L1, CD31, and D2-40 markers. A baseline computed tomography (CT) scan showed mild aortic atherosclerotic disease. He was treated with cisplatin with concurrent radical radiotherapy initially.

**Fig 1:**
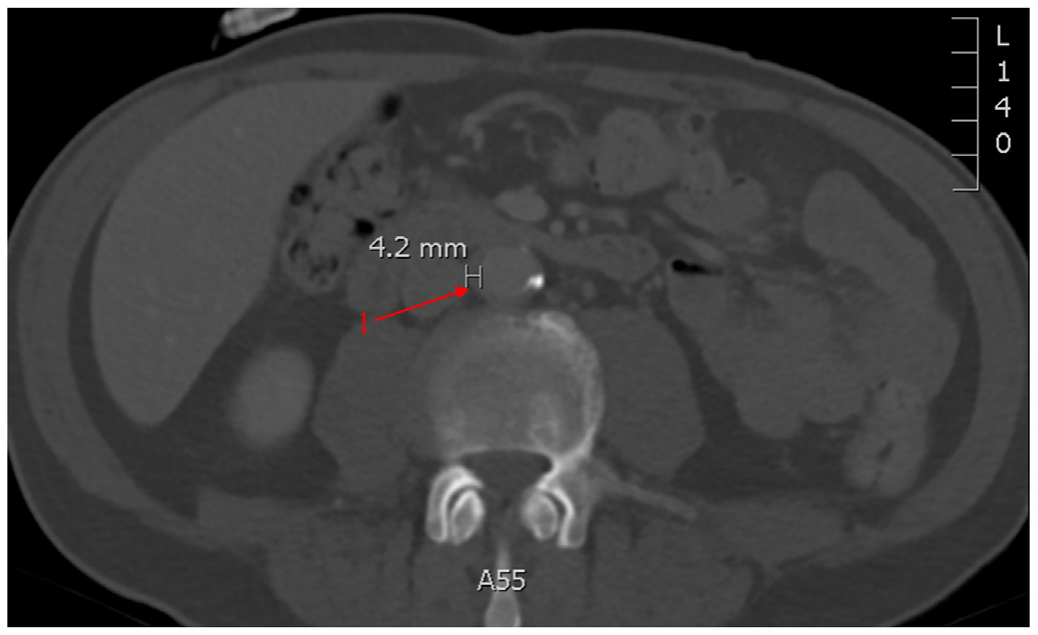
Enhanced computed tomography (CT) scan of the abdomen from May 2021. After 3 months of nivolumab therapy. Early infrarenal aortitis (1) with maximal aortic wall thickness measured at 4.2 mm. Mild aortic calcification is seen, which is stable from prior imaging.

A CT scan in December 2020 showed findings highly suggestive of pulmonary metastases. Nivolumab, an ICI, was started February 2021 with palliative intent. A CT scan in May 2021 showed improvement of the lung nodules and evidence of mild circumferential infrarenal aortic wall thickening in keeping with early aortitis changes (Fig 1). Acute phase reactants and inflammatory workup were all within normal limits and white blood cell scan was negative for infectious etiology. Repeat imaging in October 2021 and again in January 2022 demonstrated further progression with a maximal wall thickness of 0.8 cm and axial diameter measuring 3.2 cm × 2.9 cm. Owing to these progressive changes, nivolumab was held in March 2022 after discussion with medical oncology, rheumatology, and vascular surgery. The patient was asymptomatic, and the multidisciplinary team decided against steroid treatment.

**Fig 2:**
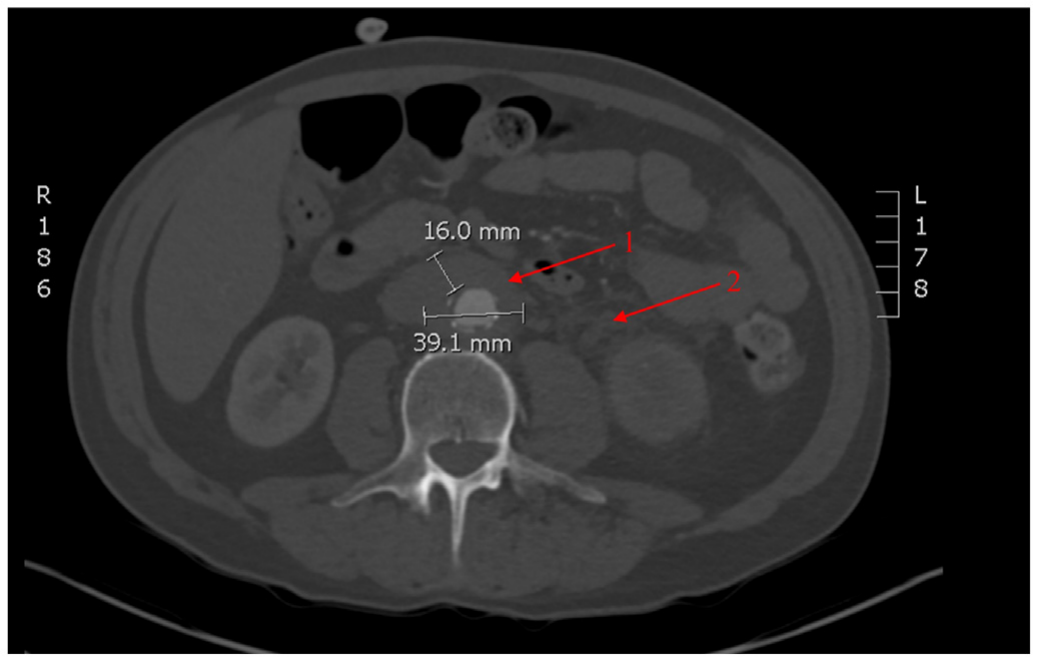
Computed tomography (CT) angiography of the abdomen from April 2022. One month after the discontinuation of nivolumab. Infrarenal aortitis (1) with maximal aortic wall thickness measured at 16 mm and axial diameter 39.1 mm. New finding of retroperitoneal fibrosis (2).

A CT scan 1 month after discontinuing nivolumab demonstrated increased maximal aortic wall thickness of 1.6 cm and axial diameter measuring 3.9 × 3.3 cm and new finding of retroperitoneal fibrosis (Fig 2). At this time, the patient remained asymptomatic. Approximately 3 weeks later, the patient presented to the emergency department complaining of intense left flank pain. A CT scan demonstrated left hydronephrosis from obstruction of the ureter, which was presumed secondary to retroperitoneal fibrosis and a ureteric stent was placed.

**Fig 3:**
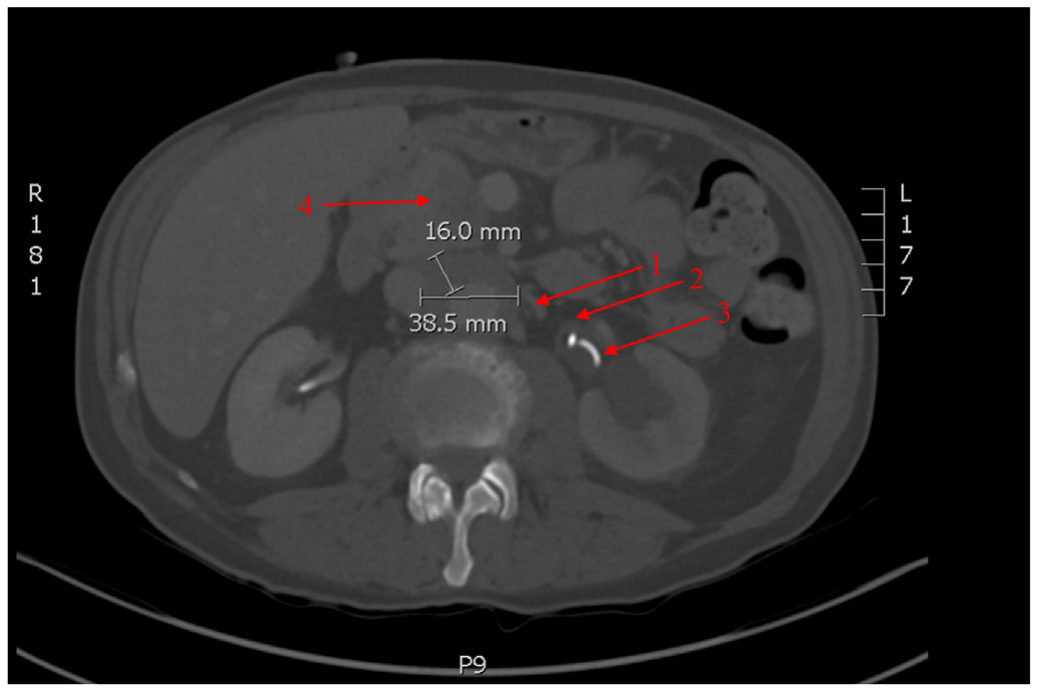
Computed tomography (CT) angiography of the abdomen from September 2022. Six months after the discontinuation of nivolumab. Infrarenal aortitis (1) with stable maximal aortic wall thickness measured at 16 mm and axial diameter 38.5 mm with persistent retroperitoneal fibrosis (2). Left ureteric stent seen (3). Additional findings of a necrotic pancreatic head mass (4) and hypodense liver lesions (not visible in this cross-section).

A follow-up CT scan 6months after discontinuing nivolumab showed stability in the infrarenal aortic measurements (Fig 3). However, the retroperitoneal fibrosis remained active and had further progressed to extend from the left renal vein to the common iliac arteries. Unfortunately, the patient developed additional findings of a necrotic pancreatic head mass and liver lesions concerning for metastases. Subsequent positron emission tomography scan showed no evidence of active aortitis, but ongoing retroperitoneal fibrosis and concerns of a pancreatic primary cancer with metastases to liver and retroperitoneal lymph nodes. The diagnosis of primary pancreatic adenocarcinoma was confirmed with fine needle aspiration. Palliative chemotherapy was initiated; however, treatment was withdrawn owing to the patient's intolerance to feeds. The patient passed away in December 2022.

## Discussion

In addition to our case, a literature search yielded 16 case reports and a case series with 3 patients (not included in table, no individual data) with ICI-associated aortitis (Table).^5^, ^6^, ^7^, ^8^, ^9^, ^10^, ^11^, ^12^, ^13^, ^14^, ^15^, ^16^, ^17^, ^18^, ^19^, ^20^, ^21^ The ICIs with the most reported cases of aortitis are the programmed cell death protein 1 inhibitors nivolumab and pembrolizumab. Other ICIs reported include programmed cell death ligand 1 inhibitors atezolizumab and durvalumab and cytotoxic T lymphocyte antigen 4 inhibitor ipilimumab. Three of these cases were also treated with pegfilgrastim, a granulocyte colony-stimulating factor, which has also been reported to have large-vessel vasculitis as a rare complication.^22^ Aortitis is diagnosed based on the presence of homogenous circumferential thickening of the aortic wall on imagining or with histopathology.^23^ Aortic biopsy is not done routinely owing to the significant risks of the procedure. Previous literature reports a diagnosis of vasculitis a median of 3 to 4 months from initiation of ICI.^21^^,^^24^ This review calculated a diagnosis of aortitis a median of 3 months from initiation of immunotherapy.

**Table:** Cases published reporting aortitis associated with immune checkpoint inhibitors (*ICIs*)

Case	Cancer	ICI	Timing, months	Clinical	Location	Treatment	Results
Present case	Head and neck	Nivolumab	3	Asymptomatic	Infrarenal aorta	Discontinue ICI	Stable aortitis, retroperitoneal fibrosis
Yildirim 2024^5^	Renal Cell	Nivolumab	33	Asymptomatic	Thoracic aorta and great vessels	Discontinue ICI, Steroid course (delayed)	Improved 6 months
Roy 2017^6^	Lung	Nivolumab	9	Back pain	Infrarenal aortic aneurysm	Discontinue ICI, Steroid course	Remission 8 weeks
Loricera 2018^7^	Melanoma	Nivolumab	2	Asymptomatic	Thoracic aorta	No data	No data
Hotta 2020^8^	Lung	Nivolumab	24	Asymptomatic	Infrarenal aortic aneurysm	Discontinue ICI	Remission 2 months
Henderson 2020^9^	Prostate	Nivolumab + ipilimumab	4	Chest and shoulder pain	Thoracic aorta and great vessels	Discontinue ICI, Steroid course	Remission 6 months
Bührer 2024^10^	Melanoma	Nivolumab + ipilimumab	16	Asymptomatic	Infrarenal aorta	Discontinue ICI, steroid course	Remission 3 months
Ohno 2024^11^	Head and neck	Nivolumab	23	Chest pain, flu-like illness	Aortic arch	Discontinue ICI, steroid course	Aneurysm rupture endovascular repair
Mort 2022^12^	Breast	Pembrolizumab + pegfilgrastim	1.5	Left shoulder and arm pain	Aortic arch and great vessels	Discontinue ICI, steroid course	Remission 8 weeks
Ninomiya 2022^13^	Lung	Pembrolizumab	2	Flu-like illness	Ascending aorta	Steroid course, then restarted ICI	Aneurysmal degeneration surgical repair
Bloomer 2022^14^	Melanoma	Pembrolizumab	15	Chest pain, flu-like illness	Aortic arch	Discontinue ICI, steroid side effects, Tocilizumab	Remission, unknown timing
Khan 2024^15^	Melanoma	Pembrolizumab	3	Asymptomatic	Ascending aortic aneurysm	Discontinue ICI	Aneurysmal degeneration surgical repair
Pinkston 2016^16^	Melanoma	Pembrolizumab	1	Abdominal pain	Perivisceral aorta	No data	No data
Liguori 2021^17^	Pancreatic	Atezolizumab	2	Abdominal pain	Perivisceral aorta	Steroid course, then restarted ICI	Recurrence treated with steroids, restart ICI with second recurrence, switch to different ICI no recurrence
Shiraha 2024^18^	Lung	Atezolizumab + pegfilgrastim	2	Back pain	Thoracic aorta and great vessels	Steroid course, then ICI restarted	Remission 3 months
Ito 2023^19^	Lung	Durvalumab + pegfilgrastim	12 days	Flu-like illness	Aortic arch	Discontinue ICI (hepatitis B, no steroids)	Remission 1 month
Ban 2017^20^	Melanoma	Ipilimumab	2	Abdominal pain, flu-like illness	Thoracic aorta	Discontinue ICI, steroid course	Symptom relapse treated with prolonged steroid taper

The management of ICI irAEs is challenging owing to a lack of standard guidelines and evidence-based recommendations, which vary based on affected organ system, severity, and clinical judgment.^25^ These recommendations include discontinuation of ICIs and initiation of high-dose steroids (prednisone or methylprednisolone 1-2 mg/kg/d), which is tapered over 4 to 6 weeks.^25^ Despite these recommendations, there is little to no evidence to support whether steroid treatment is effective; both responders and nonresponders to steroids have been reported.^24^ From the case reports, 11 patients received steroids, of which there were 2 steroid nonresponders^11^^,^^20^ and 8 steroid responders.^5^^,^^6^^,^^9^^,^^10^^,^^12^^,^^13^^,^^17^^,^^18^ Bloomer et al^14^ used tocilizumab as a steroid-sparing agent for their patient who was having side effects, and they had remission of the aortitis. Ban et al^20^ commented that a prolonged steroid taper may be necessary with severe immune reactions to achieve full response. Ohno et al^11^ treated with steroids but still had aneurysmal degeneration and rupture requiring repair. Of the three cases that did not receive steroids, two went into remission^8^^,^^19^ and one had aneurysmal degeneration requiring repair.^15^ Furthermore, it remains unclear when or if at all ICIs should be resumed after effective steroid treatment; three of the cases reinitiated ICIs after the steroid course. Ninomiya et al^13^ restarted the ICI and there was aneurysmal degeneration requiring surgical repair. Liguori et al^17^ restarted the ICI and there was recurrence of symptoms and extension of the aortitis, which was treated successfully with another course of steroids. Shiraha et al^18^ reported no recurrence with the reinitiation of ICI. Treating asymptomatic patients with steroids is also unclear. Four cases in the series were asymptomatic. Two received steroids and experienced remission.^5^^,^^10^ Two cases did not receive steroids, one of which experienced remission^8^; the other had aneurysmal degeneration requiring repair.^15^

In our patient's case, treatment with steroids may have prevented the progression of retroperitoneal fibrosis, which caused ureteric obstruction. However, at the time of the multidisciplinary meeting, the patient was asymptomatic. From the gathered cases, it seems that discontinuation of the ICI and steroids is the most common strategy and is effective at achieving remission. Even in patients who are asymptomatic, steroids may prevent the progression of complications such as aneurysmal degeneration and rupture and prevent further interventions. However, these conclusions are limited by the low case numbers. Steroids are not benign, having numerous adverse effects; therefore, the decision to give steroids should be based on multidisciplinary discussion and patient presentation.^26^

## Conclusions

Although rare, our literature search found 19 cases of aortitis in patients receiving ICIs; therefore, it is imperative to keep vascular-related irAEs in mind when prescribing for patients with cancer. As the indications for immunotherapy expand, these rare complications will become more frequent, and more data will be needed to guide their management. An appropriate strategy for treatment includes discontinuing the ICI and considering steroids. In the absence of sufficient literature, physicians must rely on multidisciplinary discussion and weighing the severity of the aortitis against the treatment of the cancer and potential side effects from steroids.

## Funding

None.

## Disclosures

None.
